# A comparison of the responsiveness of EQ-5D-5L and the QOLIE-31P and mapping of QOLIE-31P to EQ-5D-5L in epilepsy

**DOI:** 10.1007/s10198-017-0928-0

**Published:** 2017-09-04

**Authors:** Ben F. M. Wijnen, Iris Mosweu, Marian H. J. M. Majoie, Leone Ridsdale, Reina J. A. de Kinderen, Silvia M. A. A. Evers, Paul McCrone

**Affiliations:** 1 Department of Health Services Research, CAPHRI School of Public Health and Primary Care, Maastricht University, P.O. Box 616, 6200 MD Maastricht, The Netherlands; 2 Department of Research and Development, Epilepsy Centre Kempenhaeghe, Heeze, The Netherlands; 3 King’s Health Economics (KHE), Institute of Psychiatry, Psychology and Neuroscience at King’s College London, London, UK; 4 Department of Neurology, Academic Centre for Epileptology, Epilepsy Centre Kempenhaeghe and Maastricht University Medical Centre, Maastricht, The Netherlands; 5 School of Mental Health and Neuroscience, Maastricht University Medical Center, Maastricht, The Netherlands; 6 School of Health Professions Education, Faculty of Health, Medicine and Life Sciences, Maastricht University, Maastricht, The Netherlands; 7 Department of Basic and Clinical Neuroscience, Institute of Psychiatry, Psychology and Neuroscience at King’s College London, London, UK; 8 Trimbos Institute, Netherlands Institute of Mental Health and Addiction, Utrecht, The Netherlands; 9 Duboisdomein 30, 6229 GT Maastricht, The Netherlands

**Keywords:** Mapping, Responsiveness, Quality of life, Epilepsy, D610 Allocative Efficiency, Cost-Benefit Analysis

## Abstract

**Objective:**

To investigate the responsiveness of and correlation between the EQ-5D-5L and the QOLIE-31P in patients with epilepsy, and develop a mapping function to predict EQ-5D-5L values based on the QOLIE-31P for use in economic evaluations.

**Methods:**

The dataset was derived from two clinical trials, the ZMILE study in the Netherlands and the SMILE study in the UK. In both studies, patients’ quality of life using the EQ-5D-5L and QOLIE-31P was measured at baseline and 12 months follow-up. Spearman’s correlations, effect sizes (EF) and standardized response means (SRM) were calculated for both the EQ-5D-5L and QOLIE-31P domains and sub scores. Mapping functions were derived using ordinary least square (OLS) and censored least absolute deviations models.

**Results:**

A total of 509 patients were included in this study. Low to moderately strong significant correlations were found between both instruments. The EQ-5D-5L showed high ceiling effects and small EFs and SRMs, whereas the QOLIE-31P did not show ceiling effects and also showed small to moderate EFs and SRMs. Results of the different mapping functions indicate that the highest adjusted *R* ^2^ we were able to regress was 0.265 using an OLS model with squared terms, leading to a mean absolute error of 0.103.

**Conclusions:**

Results presented in this study emphasize the shortcomings of the EQ-5D-5L in epilepsy and the importance of the development of condition-specific preference-based instruments which can be used within the QALY framework. In addition, the usefulness of the constructed mapping function in economic evaluations is questionable.

**Electronic supplementary material:**

The online version of this article (doi:10.1007/s10198-017-0928-0) contains supplementary material, which is available to authorized users.

## Introduction

Epilepsy is a disorder of the brain, characterized by recurrent seizures. Seizure episodes are a result of excessive electrical discharges in a group of brain cells. Different parts of the brain can be the site of such discharges. These discharges result in a variety of clinical manifestations, depending on where they occur in the brain. The clinical manifestations can vary from the briefest lapses of attention or muscle jerks to severe and prolonged convulsions [1].

In economic evaluation, both in general and in the field of epilepsy, the quality adjusted life year (QALY) is routinely used as a summary measure of health outcome for economic evaluations, which incorporates the impact on both the quantity and quality of life (QoL). For example, the use of QALYs is required by the National Institute for Health and Clinical Excellence (NICE) in England and Wales [2] and the Healthcare institute in the Netherlands [3] for an intervention to be reimbursed. The utility part of QALYs requires health state values as QALYs are calculated based on the time spent in a specific health state multiplied by the corresponding utility of that health state. Commonly used measures to include in the QALYs are generic utility measures, such as the EuroQol 5 dimensions 5 levels (EQ-5D-5L) [4, 5], Short Form 6 dimensions (SF-6D) [6] and the Health Utility Index (HUI) [7]. Generic utility instruments are designed to be applicable in a large variety of conditions.

However, there are instances, especially in clinical research, where some generic utility measures fail to capture changes that, even if small, are important to patients. Some studies use condition-specific or condition-specific utility measures to address this limitation. It is suggested that these instruments are likely to be more responsive than generic instruments, whose strengths include breadth and applicability across conditions and interventions [8]. The responsiveness of an instrument is likely to be dependent on several factors such as the nature of the condition and the domains included in the instrument. For example, the EuroQol-5D-3L (i.e. a generic utility instrument) has been demonstrated to correlate in a moderately to good way with criterion measures in patients with chronic low back pain [9]. In contrast, the EQ-5D-3L was deemed unsuitable for people with dementia, leading to the development of a condition-specific questionnaire (DEMQOL) [10]. In epilepsy, the EQ-5D-3L has been shown to correlate well with another generic quality of life instrument, the 15D-instrument [11]. However, in patients with newly diagnosed focal epilepsy, the EuroQol-5D-3L was compared to an epilepsy-specific instrument (NEWQOL-6D) and was shown to be less responsive than the NEWQOL-6D [12]. Selai et al. [13] examined the use of the EQ-5D-3L in people with epilepsy and concluded that adaptation, seizures, and the stigma of epilepsy considerably impair quality of life but are not captured using the EQ-5D-3L, which limit its applicability [13].

Wiebe et al. [8] evaluated 43 randomized controlled trials which used generic and specific QoL instruments and concluded that specific instruments are more responsive than generic tools. Furthermore, they stress that investigators may come to misleading conclusions by using generic instruments. However, condition-specific measures lack cross-program comparability. Furthermore, if a condition-specific quality of life instrument were used for the calculation of QALYs, the valuation set should be constructed according to the same principles as generic utility measures (i.e. the multi attribute utility theory [14]), which is often not the case. An alternative option would be to derive well-conducted and validated mapping functions to map condition-specific outcomes to generic utilities. A mapping function is a regression equation used to predict values of, in this case, a generic utility instrument, using scores/values from a condition-specific instrument as regressors (also known as ‘cross-walking’) [15]. Albeit not resolving issues regarding insensitivity of generic instruments, mapping is a solution which enables health state utilities to be predicted when no preference-based measure has been included in the study [15, 16]. Such mapping functions are supposed to yield utility values comparable generic instruments [16]. However, the performance of a mapping function is dependent on and requires a degree of overlap between both measures and that the two measures are administered on the same population [15, 17]. The aim of this study is to compare the EQ-5D-5L and an often used condition-specific QoL instrument, the Quality of Life in Epilepsy-Patients-Weighted 31p (QOLIE-31P) [18]. The objective of this study is to investigate the correlation between and the responsiveness of the EQ-5D-5L and the QOLIE-31P in patients with epilepsy. In addition, we aim to develop a mapping function to predict EQ-5D-5L values based on the QOLIE-31P for use in economic evaluations.

## Methods

### QOLIE-31p

The QOLIE-31-P is a condition-specific QoL instrument which consists of 38 items assessing 7 domains of epilepsy: seizure worry, overall QOL, emotional well-being, energy-fatigue, cognitive functioning including memory, medication effects, social functioning and an overall score. In addition, for each domain, questions regarding how much distress a person feels about problems and worries related to epilepsy are included. Each domain is scored on a scale ranging from 0 to 100. Afterwards a final score can be calculated using weights derived from the amount of distress related to each domain. The final score ranges from 0 to 100, in which higher values indicate a better QoL [19].

### EQ-5D-5L

The EQ-5D-5L is generally used as a generic QoL instrument which consists of five dimensions: mobility, self-care, usual activities, pain/discomfort, anxiety/depression, each of which can have one of five responses [4, 5] (e.g. no pain, slight pain, moderate pain, severe pain and extreme pain). This measure produces a possible 3125 distinct health states ranging from 11111 (full health) to 55555 (worst). The EQ-5D-5L was valued using both the Dutch and the UK tariffs [5, 20, 21].

### Data set

The SMILE study data [22, 23] and the ZMILE study data [24] were used for the analyses. Both studies examined the (cost-) effectiveness of a self-management program for patients with epilepsy. Follow-up data was available for 12 months in both studies. Inclusion criteria for both studies were similar (i.e. epilepsy diagnosis, prescribed antiepileptic drugs, no severe psychiatric disorders, being able to participate and benefit from group sessions). However, the SMILE study included patients from age ≥16 years whereas the ZMILE study included patients aged ≥18 years, and patients in the SMILE group were also screened to have had at least two seizures in the 12 months before inclusion. Patients with complete data for each of the measures across each time point were included.

For the direct response mapping, the data set was randomly split (using the “approximately 50% of the cases” function in SPSS) into two separate data sets: (1) the “estimation sample” (*N* = 283), which was used to derive the mapping functions; (2) the “validation sample” (*N* = 224) which was used to validate the mapping functions.

### Responsiveness analyses

Descriptive analyses are presented for patient characteristics. To measure concurrent validity (i.e. the strength of the relationship between measures of the same concept) Spearman’s correlation was calculated between the domains and total scores (i.e. utilities) of the EQ-5D-5L and the QOLIE-31P. Spearman’s correlation was used due to the skewed nature of the data, especially EQ-5D-5L utilities. Strong correlations indicate that the preference-based measures are assessing related constructs. Correlations are considered weak if scores are less than 0.3, moderate if scores are between 0.3 and less than 0.7, and strong if scores are 0.7 or higher [25]. Bonferroni correction was applied to account for multiple testing (i.e. adjustment of* p*-values) [26].

To determine the predictive validity or responsiveness of both instruments (i.e. the ability of an instrument to detect relevant changes in QoL over time) the standardized response mean (SRM) and effect size (EF) were calculated. The SRM is a standard indicator of change across measures and time points and was calculated by SRM = (M1 − M2)/(SD1 − SD2), where M1 is the mean pre-assessment and M2 is the mean post-assessment, and SD1 and SD2 are the standard deviations of both assessments [25]. SRMs of less than 0.2 are considered small, 0.5 moderate, and 0.8 large [25, 27]. The EF is calculated as the difference between follow-up and baseline divided by the standard deviation of the group’s baseline scores. The SRM and EF were calculated for those patients amongst whom a change in health state was observed between baseline and follow-up. Floor and ceiling effects were examined. For each questionnaire the proportion of respondents with a minimum score (referred to as ‘floor effects’) or a maximum score (referred to as ‘ceiling effects’) was calculated. If a large proportion of the population is at the floor (lowest possible score) or ceiling (highest possible score), then this impairs the ability of the measure to pick up decreases or increases in QoL, respectively [12]. The EF and the SRM are the most common measures for responsiveness. Positive values reflect (standardized) improvements in the number of standard deviations of the baseline scores (EF) or the score differences (SRM) (i.e. unit-free) [28].

### Mapping approach

To estimate EQ-5D-5L utilities based on the QOLIE-31P, direct response mapping was used to regress QOLIE-31P scores to EQ-5D-5L utilities. In direct mapping, a regression equation is used to predict the values of the EQ-5D-5L using scores/values from the QOLIE-31P as regressors. Next, the coefficients of the model are used to carry out the conversion from the source measure to the target measure in the required dataset [29]. Spearman’s correlations of the independent variables were used to determine whether there was collinearity between independent variables, which would then be removed from the analyses. A collinearity threshold of >0.70 was used [30].

Ordinary least-squares (OLS) and censored least absolute deviations (CLAD) regression was used to estimate the model. The OLS is the most commonly used model in mapping studies [15, 31]. However, it is unable to restrict for the range of values and may lead to implausible predicted values (e.g. EQ-5D values above 1). The CLAD model was therefore used as it has the ability to account for censored or bounded data. In addition, it is robust to heteroscedasticity and can also be used for skewed data [15, 32].

As the aim of this study was to derive a predictive model, all items (domains) were included in the model despite their significance level, which is often considered best practice [33–35].

Furthermore, no attempt was made to predict the individual EQ-5D-5L dimensions separately as this has been shown to be a less efficient strategy or to give similar results in terms of prediction [36].

For both the OLS and the CLAD model, specifications of the mapping functions were constructed as proposed by Brazier et al. (2010) [15]. We started with a simple additive model by predicting EQ-5D values from the total QOLIE-31P scores including age, gender, employment, and living arrangements (model 1). Next, the EQ-5D-5L values were predicted from the 7 QOLIE-31P dimension sub scores (model 2). To relax the assumptions of the simple additive model, squared terms for dimension sub scores were included in the model (model 3) [15]. As suggested by Brazier et al. (2010) only significant squared terms were included in the model to reduce the number of variables [15].

The predictive validity of the mapping models was assessed by using: (1) the goodness of fit as assessed using adjusted/pseudo R-squared (OLS and CLAD) in the estimation sample; and (2) the predictive performance of the models in the validation sample was assessed using the mean absolute error (MAE).

All analyses were done in STATA 15 (StataCorp, College Station, TX, USA).

## Results

The dataset consisted of 509 patients of which 102 patients were recruited for the ZMILE study and 407 for the SMILE study. In total, 53.0% of the patients were female and the majority of the patients were aged between 25 and 44 years old. Most of the patients had a household or lived with others (73.1%) and 51.3% of the patients were unemployed. Mean quality of life according to the EQ-5D-5L was 0.86 and mean condition-specific quality of life according to the QOLIE-31P was 65.82. More detail regarding the characteristics of the population(s) is reported in Table 1.

**Table 1:** (Baseline) characteristics of the population

Characteristics	ZMILE sample	SMILE sample	Total
	(*n* = 102)	(*n* = 407)	(*n* = 509)
Gender			
Male	50 (49.0%)	185 (45.5%)	235 (46.2%)
Female	52 (51.0%)	219 (53.8%)	271 (53.2%)
Missing values	0	3	
Age in years			
16–24	17 (16.7%)	46 (11.3%)	63 (12.4%)
25–44	42 (41.2%)	194 (47.7%)	236 (46.4%)
45–64	37 (36.2%)	142 (34.9%)	179 (35.2%)
≥65	6 (5.9%)	22 (5.4%)	28 (5.5%)
Missing values	0	3	
Living arrangements, *n* (%)			
Household/living with others	67 (65.7%)	305 (74.9%)	372 (73.1%)
Living alone	26 (25.5%)	95 (23.4%)	121 (23.8%)
Other arrangements	4 (3.9%)	4 (1%)	8 (1.6%)
Missing values	5	3	
Employment			
Not unemployed	54 (52.9%)	207 (50.9%)	261 (51.3%)
Specifically employed or student	44 (43.1%)	197 (48.4%)	241 (47.4%)
Missing values	4	3	
Quality of life			
EQ-5D-5L baseline	0.83	0.87	0.86
EQ VAS	74.77	67.00	68.53
QOLIE-31P baseline	64.74	66.05	65.82

### Validity and responsiveness

An assessment of the strength of the relationship between the EQ-5D-5L and the QOLIE-31P based on Spearman’s correlation coefficient, showed moderately strong significant correlations between both instruments for the total score (Table 2). Only a few statistically significant correlations were found between the sub scores of the QOLIE-31P and the sub scores of the EQ-5D-5L. All sub scores of the QOLIE-31P did significantly correlate with the total EQ-5D-5L scores. At baseline and 12 months follow-up ceiling effects on the EQ-5D-5L were substantial with 37.8 and 33.5% of the patients reporting the maximum score. No ceiling effects were found for the QOLIE-31P.

**Table 2:** Spearman’s correlation coefficients between QOLIE-31P values and EQ-5D-5L values

	EQ-5D mobility	EQ-5D self-care	EQ-5D usual activities	EQ-5D pain	EQ-5D anxiety and depression	Total EQ-5D score
QOLIE-31P energy	−0.2330	−0.1896	−0.1991	−0.2689*	−0.2293	0.4499*
QOLIE-31P mood	−0.1252	−0.0285	−0.1725	−0.1743	−0.3823*	0.4881*
QOLIE-31P daily activities	−0.2350	−0.2138	−0.2557*	−0.2644*	−0.2422*	0.4335*
QOLIE-31P cognition	−0.1041	−0.0607	−0.1906	−0.1661	−0.2539*	0.2948*
QOLIE-31P medication effects	−0.0569	−0.1061	−0.1744	−0.1969	−0.2304	0.2730*
QOLIE-31P seizure worry	−0.1099	−0.0802	−0.1502	−0.1901	−0.3057*	0.3906*
QOLIE-31P overall QOL	−0.1794	−0.1620	−0.2245	−0.1808	−0.3142*	0.4049*
Total QOLIE-31P score	−0.2000	−0.1476	−0.2855*	−0.3171*	−0.3354*	0.5653*

* Significant correlation at 5% level

Details regarding the EF and SRM are presented in Table 3. EFs and SRMs all appear to be relatively small. Both the EF and SRM estimates are smaller for the EQ-5D-5L than the QOLIE-31P. For the EQ-5D-5L, values range from −0.017 to 0.043 for the EF and from −0.023 to 0.025 for the SRM which would be considered small. The EF and SRM values for the QOLIE-31P range from 0.082 to 0.290 (EF) and from 0.07 to 0.270 (SRM), which would be regarded as small to moderate.

**Table 3:** Standardized response means for QOLIE-31P and EQ-5D-5L

	Mean difference between BS and FU12M	Effect size	Standardized response mean
Total EQ-5D-5L score	−0.004	−0.017	−0.023
EQ-5D mobility	0.276	0.010	0.003
EQ-5D self-care	0.305	0.010	0.003
EQ-5D usual activities	2.600	0.043	0.025
EQ-5D pain	0.619	0.012	0.006
EQ-5D anxiety	−1.847	−0.017	−0.019
Total QOLIE-31P score	2.414	0.187	0.212
QOLIE-31P energy	1.875	0.110	0.080
QOLIE-31P mood	1.906	0.082	0.070
QOLIE-31P daily activities	8.317	0.284	0.270
QOLIE-31P cognition	3.196	0.131	0.125
QOLIE-31P medication effects	5.878	0.177	0.178
QOLIE-31P seizure worry	7.696	0.290	0.254
QOLIE-31P overall QOL	2.963	0.128	0.116

*EQ-5D-5L* EuroQol 5 dimensions with 5 levels,* BS* baseline,* FU12M* follow-up measurement at 12 months

### Mapping functions

The EQ-5D index scores had a somewhat bimodal distribution, and the distribution of the QOLIE-31P index scores were normally distributed (see Online Supplementary Materials 1). The inclusion of age was shown to have a significant effect on the prediction of EQ-5D-5L scores. All other demographic variables were excluded from the analyses. In addition, there was a significant effect associated with country (i.e. SMILE or ZMILE dataset). Hence, age and country were included in all mapping functions.

**Fig. 1:**
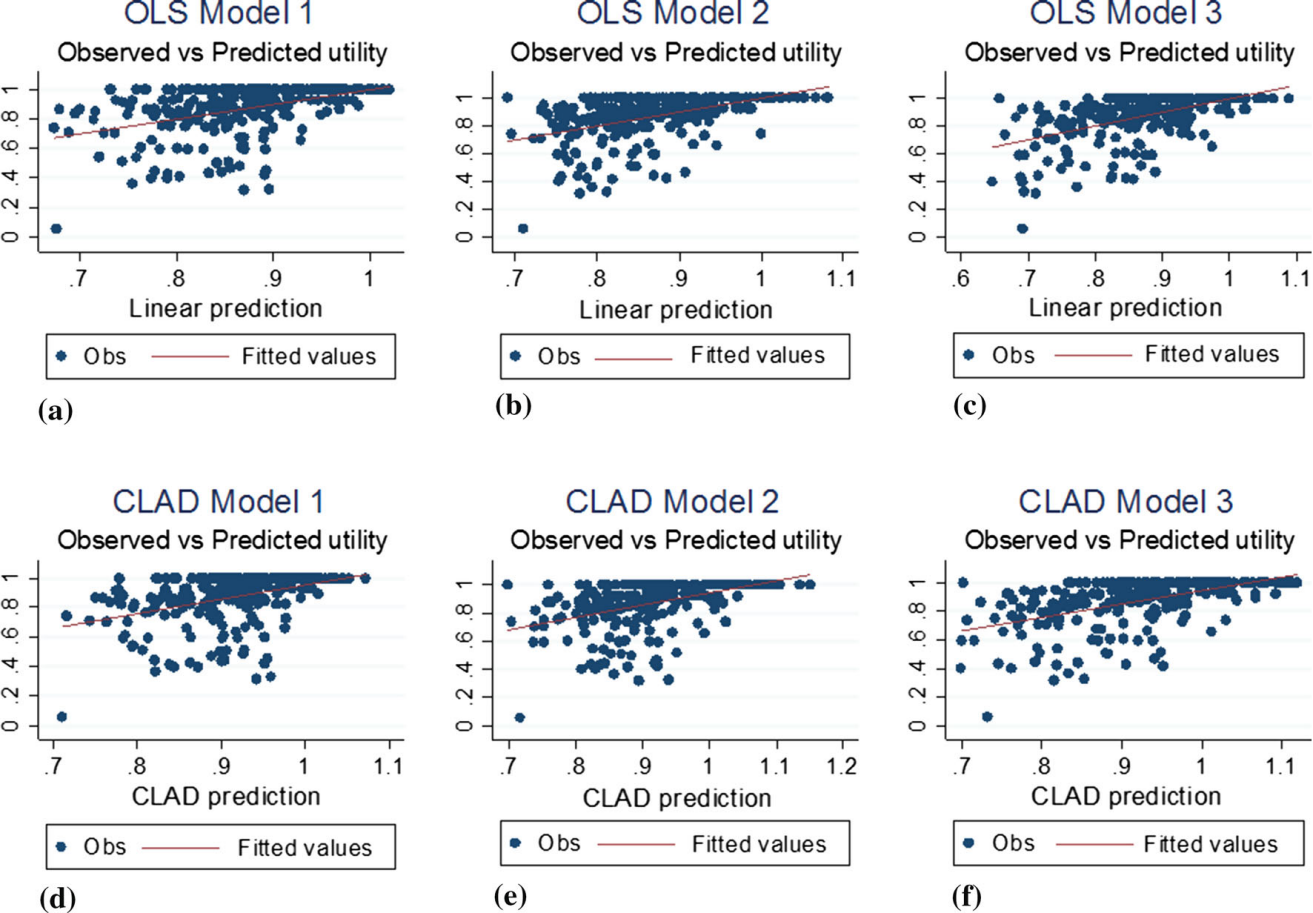
Scatter plots comparing observed vs predicted EQ-5D-5L values for OLS (**a**–**c**), CLAD (**d**–**f**)

For the OLS mapping functions, model 3 performed best with an MAE of 0.103 and an adjusted *R* ^2^ of 0.265. Inclusion of age significantly improved the model, hence a model without age was only constructed for OLS. For the CLAD mapping functions, CLAD model 3 performed best with a MAE of 0.097 and a pseudo *R* ^2^ of 0.160. It should be noted, however, that including squared terms only improved adjusted/pseudo *R* ^2^ values and only marginally improved MAE in the estimation sample (see Table 4). All models predicted values above 1 (full health), of which OLS model 2 was closest to 1 with maximum values of 1.020. A graphical representation of the model fits is presented in Fig. 1.

**Table 4:** Summary of observed and predicted values for all models in estimation dataset (*N* = 283)

	Observed EQ-5D utility	Predicted EQ-5D utilities			
		Total QOLIE-31P scores	Total QOLIE-31P and age and country	QOLIE-31P domain scores and age and country	Domain scores and squared terms and age and country
		OLS model 0	OLS model 1	OLS model 2	OLS model 3
Mean	0.867	0.868	0.868	0.871	0.872
Minimum	0.055	0.668	0.673	0.690	0.647
Maximum	1.000	1.026	1.020	1.082	1.086
MAE		0.110	0.107	0.103	0.103
Adjusted *R* ^2^		0.151	0.180	0.211	0.265
			CLAD model 1	CLAD model 2	CLAD model 3
Mean	0.867	–	0.915	0.916	0.920
Minimum	0.055	–	0.711	0.697	0.698
Maximum	1.000	–	1.072	1.15	1.119
MAE		–	0.099	0.099	0.097
Pseudo *R* ^2^		–	0.116	0.129	0.160

*MAE* mean absolute error, *OLS* ordinary least squares, *CLAD* censored least absolute deviations

When assessing the MAE in the validation sample, using the mapping functions derived from the estimation sample, OLS model 1 performed best with a MAE of 0.114. Likewise, for the CLAD mapping functions, model 1 performed best with a MAE value of 0.109 (see Table 5). Given the higher adjusted/pseudo *R* ^2^ values of model 3 compared to model 1 for both OLS and CLAD, and given the lower MAE for OLS model 3 compared to CLAD model 3 in the validation sample, the best mapping function would be OLS model 3. The regression coefficients for this model are presented in Online Supplementary Materials 1.

**Table 5:** Summary of observed and predicted values for all models in validation dataset (*N* = 224)

	Observed EQ-5D utility	Predicted EQ-5D utilities		
		Total QOLIE-31P and age and country	QOLIE-31P domain scores and age and country	Domain scores and squared terms and age and country
		OLS model 1	OLS model 2	OLS model 3
Mean	0.863	0.865	0.865	0.865
Minimum	0.054	0.649	0.685	0.629
Maximum	1.000	1.039	1.100	1.084
MAE		0.114	0.116	0.118
		CLAD model 1	CLAD model 2	CLAD model 3
Mean	0.863	0.912	0.907	0.911
Minimum	0.054	0.682	0.688	0.699
Maximum	1.000	1.062	1.136	1.120
MAE		0.109	0.117	0.119

## Discussion

The aim of this study was to compare the responsiveness of the QOLIE-31P and the EQ-5D-5L in epilepsy and to predict EQ-5D-5L values based on QOLIE-31P scores with the development of a mapping function. Looking at concurrent validity, although the correlations were significant, the strength was only moderate between both instruments when looking at the total score. This may imply that both instruments are only measuring the same concept to some extent and impose conceptual differences.

The EQ-5D-5L showed substantially high ceiling effects and was demonstrated to have rather small EFs and SRMs, whereas the QOLIE-31P did not show ceiling effects and had small to moderate EFs and SRMs. This indicates that the QOLIE-31P has an overall higher responsiveness based on EF, SRM and ceiling effects. Furthermore, this study provides a mapping function which can be used in (future) economic evaluations to map QOLIE-31P data to EQ-5D-5L values.

The relatively small EFs and SRMs may be explained by a lack of responsiveness of both instruments, especially the EQ-5D-5L. However, part of these small estimates can be explained by the fact that the patients’ health state did not change much over time. The EF and SRM were calculated in all patients who had any change during follow-up, due to the lack of a known clinically meaningful difference for both instruments; this led to an underestimation of the EF and SRM. The EQ-5D-5L, however, performed substantially worse than the QOLIE-31P (i.e. lower estimated EFs and SRMs).

Using different mapping functions, the highest adjusted *R* ^2^ we were able to regress was 0.265 using an OLS model with squared terms, which led to a MAE of 0.103. Overall, this model performed best given the results within the estimation and validation sample. Although theoretically preferred, the use of a CLAD model did not perform better than the OLS model, especially in the validation sample. Mixed results have been reported in studies using CLAD models [17], with some concluding that CLAD improved the model fit [37, 38] and others concluding that the improvement of CLAD over OLS was small or did not have an impact [39]. The adjusted/pseudo *R* ^2^ values found in this study were relatively small, which is not uncommon. In a review of Brazier et al. (2010), it was found that models mapping a generic instrument onto a generic preference-based measure achieved an adjusted *R* ^2^ of more than 0.5 within sample. However, in studies examining the fit of functions mapping from condition-specific to generic measures, results were more variable ranging from 0.17 to 0.51 [15]. In addition, errors were often larger for models mapping a generic measure onto a generic preference-based measure [15, 17]. Likewise, the usefulness of our mapping function in economic evaluations is questionable given the relatively large mean absolute error and poor model fit.

Another way of mapping would be to use a model to predict responses of each of the five dimensions of the EQ-5D-5L from the QOLIE-31P (sub) scores; so-called indirect response mapping or response mapping models [40]. As the purpose of the mapping part of this study was to derive a regression function, this method was not applied. In addition, as mentioned above, indirect response mapping has been shown to be a less efficient strategy or to give similar results in terms of prediction [36].

The use of mapping to derive EQ-5D-5L values is fundamentally limited by the degree of overlap between two instruments [17]. Although several studies reported limitations with generic preference-based quality of life instruments regarding their responsiveness and ability to discriminate between health states (e.g. McTaggart-Cowan et al. [41]), the use of generic preference-based instruments is mandatory in most national guidelines for pharmacoeconomic evaluations, for example in the UK and the Netherlands [3, 42]. However, given the limited responsiveness, low correlations, and the poor model fit of the mapping functions it may be argued that there is a need for the development of condition-specific preference-based measures for patients with epilepsy. General (non-preference based) condition-specific instruments, such as the QOLIE-31P, are an important source of evidence; however, their use in economic evaluation is severely limited because they were not designed for this purpose and, unless they are preference-based, they theoretically cannot be used to calculate quality adjusted life years (QALYs) [14, 43]. Several attempts have been made to derive condition-specific quality of life instruments to calculate (condition-specific) QALYs, such as the development of new instruments [44, 45] or the development of condition-specific preference-based measures from existing instruments [46, 47]. Of course, such a measure could not be the sole outcome of interest for economic evaluations, as they lack the comparability between conditions, a distinct advantage of generic instruments [48]. We agree with Brazier et al. (2010), that development of a condition-specific preference-based instrument should not be seen as an alternative to generic preference-based measures, but rather as a supplement [43]. Condition-specific preference-based instruments may have an important role in ensuring that the benefits of health-care interventions are adequately reflected in QALY estimates for economic evaluations in all conditions [49].

This study is subject to several limitations. First, we only investigated the use of a few mapping models, whereas a wide variety of models exist, such as GLM or Tobit models. Furthermore, other correlation coefficients may have been used, such as polychoric correlation coefficients. However, given the marginal differences between the models used in this study, the model fit is not likely to be improved substantially. In addition, structural equation modelling could be used to analyze the structural relationship between EQ-5D-5L and QOLIE-31P and latent constructs. Second, our estimations are based on a pooled data set containing data from both the UK and the Netherlands. Although inclusion criteria for both studies were similar and there was no significant difference between the countries regarding regression estimates, this may have introduced extra heterogeneity within the data. Lastly, the pooled dataset was divided into an estimation sample and a validation sample. This has the advantage that it assesses the mapping function by its prime purpose; however, it reduces the sample size of the estimation sample. The use of the whole sample for the model estimations, however, did not substantially improve the model(s).

## Conclusion

There was a low to moderate correlation between the sub scores and total scores of the EQ-5D-5L and the QOLIE-31P. Both the EF and SMRs were relatively low, especially for the EQ-5D-5L. Mapping functions to regress QOLIE-31P values to EQ-5D-5L values did not show an optimal fit with relatively low adjusted *R* ^2^ values. The results presented in this study may emphasize the importance of the development of condition-specific preference-based instruments which can be used within the QALY framework and hence be incorporated as an important supplement in economic evaluations. The development of such condition-specific preference-based quality of instruments can ensure that the benefits of health-care interventions are adequately reflected in QALY estimates for economic evaluations not only in epilepsy but for all conditions.

## Electronic supplementary material

Below is the link to the electronic supplementary material.

Supplementary material 1 (DOCX 22 kb)
