# Supplementary material for: A comparison of the responsiveness of EQ-5D-5L and the QOLIE-31P and mapping of QOLIE-31P to EQ-5D-5L in epilepsy
Source: Eur J Health Econ. 2017 Sep 4;19(6):861–70. doi: 10.1007/s10198-017-0928-0 (PMC6008365; doi:10.1007/s10198-017-0928-0)
Supplement: Supplementary file 1 — Supplementary material 1 (DOCX 22 kb) [file 10198_2017_928_MOESM1_ESM.docx]

**Supplementary Material 1**

Distribution of both EQ-5D-5L values and QOLIE-31P values

**Supplementary Material 2**

“Best” mapping function given adjusted ^R^ values and mean absolute error rates to map QOLIE-31P values to EQ-5D-5L values

|  | **Coefficients** | **Std. Error** |
| --- | --- | --- |
| (Constant) | 0.68416780 | 0.0480296 |
| Country | 0.07690210 | 0.0243357 |
| Age (years) | -0.00134930 | 0.0006669 |
| Score sub score “Seizure worry” | 0.00027910 | 0.0003756 |
| Score sub score “Overall QOL” | 0.00022530 | 0.0014603 |
| Score sub score “Emotional well-being” | 0.00431610 | 0.0014529 |
| Score sub score “Energy-fatigue” | 0.00408590 | 0.0015880 |
| Score sub score “Cognitive functioning” | -0.00009420 | 0.0003570 |
| Score sub score “Medication effects” | -0.00348630 | 0.0011060 |
| Score sub score “Social functioning” | 0.00073940 | 0.0004200 |
| Squared sub score “Energy-fatigue” | -0.00002710 | 0.0000172 |
| Squared sub score “Emotional well-being” | -0.00004190 | 0.0000143 |
| Squared sub score “Medication effects” | 0.00003180 | 0.0000102 |
| Squared sub score “Overall QOL” | 0.00000632 | 0.0000146 |
| Adjusted R^2^ | 0.265 | |
